# Supplementary material for: Coexistence rules for small, antagonistically interacting microbial communities
Source: PLoS Comput Biol. 2025 Dec 4;21(12):e1013763. doi: 10.1371/journal.pcbi.1013763 (PMC12697945; doi:10.1371/journal.pcbi.1013763)
Supplement: S1 Appendix — (PDF) [file pcbi.1013763.s001.pdf]

S1 Appendix:

Coexistence rules for small, antagonistically interacting microbial communities

Gaurav S. Athreya[a,b], Chaitanya S. Gokhale[c,d] & Prateek Verma[e,f,g*]

[a] Indian Institute of Science Education and Research, Pune, India

[b] Current Address: Institute of Organismic and Molecular Evolution (iomE), Johannes Gutenberg University, Mainz, Germany

[c] Research Group for Theoretical Models of Eco-evolutionary Dynamics, Department of Evolutionary Theory, Max Planck Institute for Evolutionary Biology, Plön, Germany

[d] Center for Computational and Theoretical Biology, Julius-Maximilians University Würzburg, Würzburg, Germany

[e] Current Address: Divisions of Epidemiology and Biostatistics, School of Public Health, University of California, Berkeley, California, United States of America

[f] Institute for Chemistry and Biology of the Marine Environment, Carl von Ossietzky University of Oldenburg, Oldenburg, Germany

[g] Faculty of Business Administration and Economics, Bielefeld University, Bielefeld, Germany

A.1 Removing redundancies in community space

This section describes how the set of all communities with N strain and M antibiotics (henceforth $\Omega_{N,M}$) can be efficiently enumerated. The mixed-inhibition zone model requires the specification of NM phenotypes, i.e. the phenotype (producer, sensitive, degrader, or resistant) of N strains, each of which with respect to M antibiotics. This implemented here by the introduction of the matrices P, S, D, and R, where e.g. $P_{ij} = 1$ if strain i produces antibiotic j, and $P_{ij} = 0$ otherwise. The other matrices S, D, R have analogous meaning. This can be condensed to one $N \times M$ matrix T, which has entries T_{ij} such that T_{ij} can take values in $\{1, 2, 3, 4\}$, corresponding to the possible phenotypes of strain i with respect to antibiotic j - producer (P), sensitive (S), degrader (D), and intrinsically resistant (R) respectively. See Figure A.1 for an example.

$$\underset{\mathbf{P}}{\begin{bmatrix}0 & 0\\0 & 1\end{bmatrix}} \quad \underset{\mathbf{S}}{\begin{bmatrix}0 & 0\\0 & 0\end{bmatrix}} \quad \underset{\mathbf{D}}{\begin{bmatrix}0 & 1\\1 & 0\end{bmatrix}} \quad \underset{\mathbf{R}}{\begin{bmatrix}1 & 0\\0 & 0\end{bmatrix}} \longrightarrow \underset{\mathbf{T}}{\begin{bmatrix}4 & 3\\3 & 1\end{bmatrix}}$$

Fig. A.1: We describe a microbial community in our model and computer scripts by a collection of 4 binary matrices P, S, D, R where the letters stand for Producer, Sensitive, Degrader, and Resistant respectively. These matrices function as follows: $P_{ij} = 1$ iff strain i is a producer of antibiotic j, $S_{ij} = 1$ iff strain i is sensitive to antibiotic j, etc.. They can be condensed into one matrix T; we show an example for N=2, M=2 of this condensation of P, S, D, R into some $T \in \mathbb{T}_{2,2}$, the set of all 2×2 matrices with entries in $\{1, 2, 3, 4\}$

We are therefore describing $\Omega_{N,M}$ by using the set $\mathbb{T}_{N,M}$ of all $N \times M$ matrices with entries in $\{1, 2, 3, 4\}$. By the assumption of 4 discrete phenotypes, the size of $\mathbb{T}_{N,M}$ is 4^{NM}, since each matrix can be produced by choosing NM numbers – the number of entries in the matrix – and there are 4 possibilities for each choice (1,2,3 or 4). It is clear that $\mathbb{T}_{N,M}$ contains all communities since any community can be described by a set of 4 matrices P, S, D, R which can then be condensed to an element of $\mathbb{T}_{N,M}$. However, $\mathbb{T}_{N,M}$ as described so far contains multiple copies of each community. The subject of this section is to describe this redundancy, as well as a method by which it can be eliminated, to improve the efficiency of our analysis.

As an instructive example, consider the case of $N = 2$ and $M = 1$, that is, the case of 2 strains and 1 antibiotic. $\mathbb{T}_{2,1}$ is of size $4^{2\times1} = 16$, but explicit computation shows that the size of $\Omega_{2,1}$ is 10, i.e., smaller than 16. Suppose we describe communities by their strain composition, and we describe each strain using an ordered string of letters from $\{$P,S,D,R$\}$ to denote the strain's phenotype with respect to each antibiotic. The communities in $\Omega_{2,1}$ are thus $\{$P,S$\}$, $\{$P,D$\}$, $\{$P,R$\}$, $\{$S,D$\}$, $\{$S,R$\}$, and $\{$D,R$\}$. The 2×1 matrices in $\mathbb{T}_{2,1}$, however, take into account

1. the difference in ordering between $\{$P,S$\}$ and $\{$S,P$\}$ (there are 6 pairs like this) - but a community containing 1 producer strain and 1 sensitive strain is the same as a community containing 1 sensitive strain and 1 producer strain, and

2. the 4 matrices of the form $\{$P,P$\}$ - but these strains are identical in all ways considered so this community must not be counted as containing two distinct species

Doing this reduction by hand becomes increasingly cumbersome (and difficult) with increasing N and M, and it is therefore useful to have a method of performing this reduction for arbitrary N and M.

We begin by noting that to interpret the matrices in $\mathbb{T}_{N,M}$ as encoding strain phenotypes of a community as above, it is necessary to fix a labelling of strains and antibiotics. It is this labelling that induces the redundancy since there is no biological significance to it. Consider for example the two pairs of matrices shown in Figure A.2. The first pair has rows interchanged - denoting a different labelling of the N strains, whereas the second pair has columns interchanged - denoting a different labelling of the M antibiotics. Despite the two operations (row and column switching) leading to a different element of

$\mathbb{T}_{2,3}$, we want the matrices before and after the operations to describe the same community. In other words, despite an arbitrary number of permutations of its rows and columns, a matrix will describe the same community. It is therefore natural to turn to the idea of graph isomorphisms since the structure of a graph is also identical under different labelings of its vertices and edges.

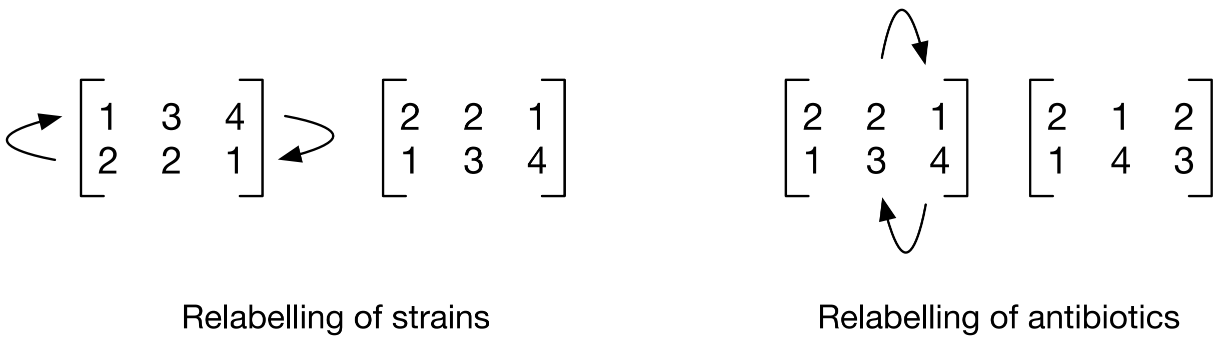

Fig. A.2: The matrix description even after condensation as in the previous figure is redundant - the identity of a matrix is not uniquely given by a matrix in $\mathbb{T}_{N,M}$. Instead, many matrices describe the same community. This is because a community does not change if the strains or antibiotics are merely relabelled - called 1 and 2 (in order) instead of 2 and 1. This order is externally imposed, and so does not change the community. This figure shows redundant description of the same community by different elements of $\mathbb{T}_{2,3}$.

Before describing a method to remove the redundancy, it is interesting to note that the number of elements in $\Omega(N, M)$ can be computed. Each community corresponds to a matrix with non-repeating rows and columns (point 2 above), and with no double-counting due to row and column permutations (point 1). The number of matrices with N non-repeating rows and M non-repeating columns is [1]

$$\sum_{i=0}^{N}\sum_{j=0}^{M} s(N,i)s(M,j)4^{ij} \tag{A.1}$$

which is derived using an inclusion-exclusion argument. This sequence is available as entry A181230 of the OEIS [2]. From these matrices, we also wish to remove all row- and column-permuted versions of a given matrix, and only keep one representative. There are $N!$ row permutations, and $M!$ column permutations, therefore the number of communities is

$$\frac{1}{N!M!}\sum_{i=0}^{N}\sum_{j=0}^{M} s(N,i)s(M,j)4^{ij} \tag{A.2}$$

We construct these communities as follows. Let T be a matrix in $\mathbb{T}_{N,M}$ and let $C \in \Omega_{N,M}$ be the community described by T. First we transform T into a labelled bipartite graph which we will call the *antibiotic profile* of T, with the two parts representing the strains and antibiotics, giving rise to a set $\mathbb{G}_{M,N}$ of bipartite graphs. We then perform a reduction on $\mathbb{G}_{M,N}$ using the notion of an isomorphism of graphs, and convert the reduced set of graphs back into matrices.

Given $T \in \mathbb{T}_{N,M}$, construct $G = (S \cup A, E)$, a labelled bipartite graph with N vertices in one part S, and M vertices in the other part A. The vertices in S denote the strains and the vertices in A denote the antibiotics. All vertices in S are connected to all vertices in A, but the (labelled) edges may have different weights. In particular, the label of the edge going from a vertex $i \in S$ to a vertex $j \in A$ is exactly the

element T_{ij}. In other words, the edge labels denote the phenotype of strain i with respect to antibiotic j. See Figure A.3 for a depiction of this operation.

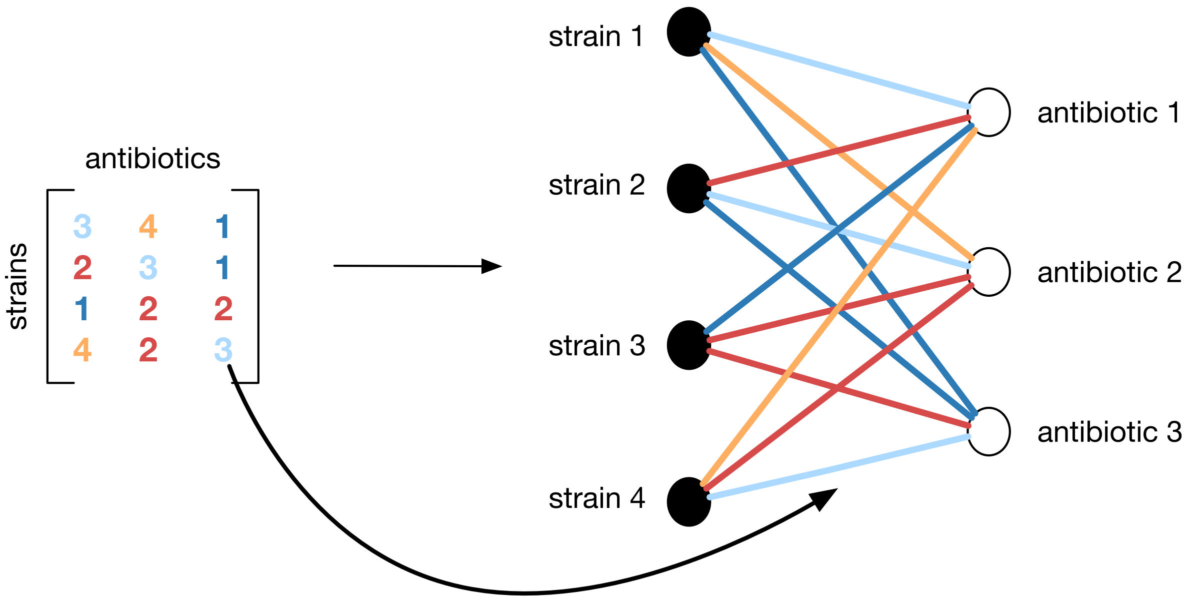

Fig. A.3: This invariance of the community under relabelling is exactly what is captured by a graph - the community is described only by the phenotype of each strain with respect to each antibiotic. We envision the matrices in $\mathbb{T}_{N,M}$ as adjacency matrices of labelled, bipartite graphs, and claim that the graphs i.e., the relative connections of strains and antibiotics, uniquely specifies a community, *but only upto isomorphism*. This figure shows an example of the transformation from $\mathbb{T}_{4,3}$ to $\mathbb{G}_{4,3}$, which is defined here and also for any N, M as the image of $\mathbb{T}_{4,3}$ under this map given by interpreting the matrices as specifying the edges of a graph. Since there are efficient algorithms to check for isomorphisms between graphs, we develop an algorithm to derive a set of non-isomorphic graphs instead of manipulating the matrices themselves, which is in principle possible. In other words, we want a set of graphs such that given any matrix in$\mathbb{T}_{N,M}$ for some N and M, there is a graph in our collection that corresponds to this matrix, perhaps after relabelling strains and antibiotics.

An *isomorphism* between 2 graphs X and Y is a one-to-one correspondence f between the vertex sets of X and Y such that any two vertices u and v of X are adjacent in X if and only if $f(u)$ and $f(v)$ are adjacent in Y, with the edges (u, v) and $(f(u), f(v))$ having same edge weights and direction. Two graphs are called isomorphic to each other if there exists an isomorphism between them. See Figure A.4 for an example of two isomorphic graphs. Intuitively, an isomorphism corresponds to a relabelling of the vertices or a re-drawing of the graph. The *isomorphism class* of a graph is the set of all graphs it is isomorphic to. Importantly, note that any two isomorphic graphs have the same isomorphism class.

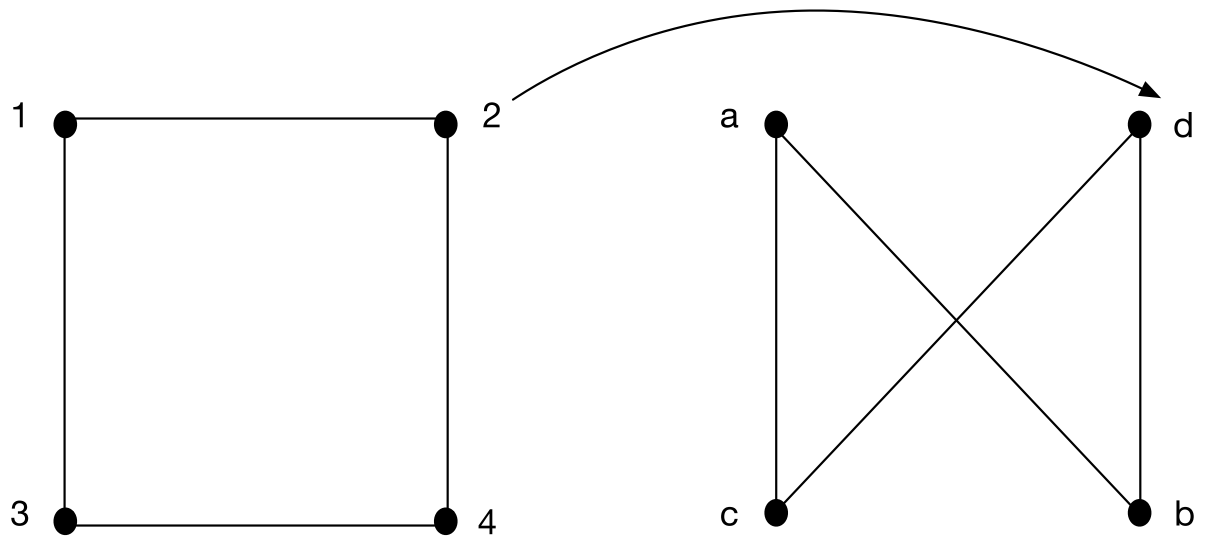

Fig. A.4: Two graphs are isomorphic if they are just re-drawings of each other - that is, if there is a correspondence of their vertices such that they look the same. This figure shows an example of two isomorphic graphs on 4 vertices. The isomorphism is as follows: $1 \to a$, $2 \to b$, $3 \to c$, $4 \to d$. Intuitively, the graph on the right is a re-drawing of that on the left since it can be obtained by twisting the right side of the square (and thus the horizontal edges as well) when one physically "flips" vertices 2 and 4 out of the plane of the page.

The remaining paragraphs describe the method by which the reduction is performed on $\mathbb{G}_{M,N}$. It is sufficient to only consider one element from the isomorphism class of each graph in $\mathbb{G}_{M,N}$. The redundancy is hence removed by discarding graphs which fall into the isomorphism class of a graph already considered, since isomorphic graphs have the same isomorphism class. The procedure is as follows:

- Initialize a storage structure $\mathcal{S}$.
- Generate all matrices in $\mathbb{T}_{N,M}$.
- Now we iterate over $\mathbb{T}_{N,M}$ - for each matrix T, convert T to the corresponding bipartite graph G as above.
- For each graph already present in $\mathcal{S}$, check if it is isomorphic to G. (this is the expensive step)
- G is discarded as soon as we encounter a graph in $\mathcal{S}$ that is isomorphic to it.
- In other words, G is added to $\mathcal{S}$ iff it is non-isomorphic to all graphs already present in $\mathcal{S}$.

All that remains is to prove that this procedure gives rise to a subset of $\mathbb{G}_{M,N}$ which is in one-to-one correspondence with the set $\Omega_{N,M}$ of all communities with N strains and M antibiotics. Recall that $\mathbb{T}_{N,M}$ already describes $\Omega_{N,M}$ fully, but redundantly. By our construction of $\mathcal{S}$, we see that no pair of graphs in $\mathcal{S}$ can be isomorphic to each other. Therefore, we need to prove that two antibiotic profiles describe the same community in $\Omega_{N,M}$ if and only if they are isomorphic.

Remark. For the more mathematically inclined, this "reduction" amounts to interpreting the matrices in $\mathbb{T}_{M,N}$ as adjacency matrices of the graphs in $\mathbb{G}_{M,N}$ and taking the quotient of $\mathbb{G}_{M,N}$ by the equivalence relation induced by graph isomorphism. Then we map the non-redundant set of graphs back to

their adjacency matrices – this map would have failed to be injective if the redundant graphs were not removed.

First suppose we construct two antibiotic profiles X, Y for the same community by adopting two (possibly different) labellings. In this case, we can explicitly construct an isomorphism between X and Y since we know the labels of each strain in both X and Y. Conversely, suppose we have two isomorphic antibiotic profiles. Both must necessarily be bipartite. We can match the strain parts and the antibiotic parts of the two graphs by looking at the vertex labels - the strain parts are the parts which have vertices labelled 'S'. We can then consult the isomorphism to infer which vertices are mapped to each other. By definition of an isomorphism, the mapped vertices will have the same edges and hence the same phenotype.

The above if and only if condition means that each matrix in the output of this algorithm represents a distinct community. In other words, our algorithm performs the maximum possible reduction in search space.

A.2 Details of the stochastic spatial model

Suppose there are N strains and M antibiotics. We initialise an $L \times L$ lattice and set the initial condition for population dynamics as follows: for each grid cell, a "spore" of type i ($i = 1, ..., N$) is placed at this grid cell with probability p, where p is chosen such that $Np < 1$. These spores then reproduce and spread over the lattice to occupy all grid cells. We drop the assumption that individuals can disperse over large distances, which differentiates this model from the well-mixed model of previous sections. One *time step* or *generation* is defined as the total time taken to update the state of each individual currently on the lattice. An *update step* is defined as the process of updating the state of a single individual.

At each time step, we iterate over all grid cells on the lattice in a random order. If the grid cell is empty, we move on to the next. If the grid cell is occupied by an individual A, there are two processes taking place - first, consider the diffusion of antibiotics and degrader chemicals. We depart from the simplifying assumption made by Kelsic et al. (2015) that antibiotics and degraders are binary in their effect, i.e., either fully effective within an area of K_P or K_D or not effective at all outside this area [3]. We assume each individual produces a fixed amount of antibiotic/degrader chemicals per update step. If A produces any antibiotics, then u_p units of each antibiotic type are placed on the focal grid cell. Similarly, if A degrades any antibiotics, u_d units of each degrader chemical are placed on the focal grid cell. Once all chemicals have been secreted, a Gaussian filter is applied independently to the concentrations of each antibiotic and each degrader molecule to simulate their diffusion. On a grid cell with a non-zero amount of antibiotic and corresponding degrader, we assume that the resultant antibiotic amount, i.e., post-inactivation by degradation, is the difference between the number of antibiotic and degrader units present in this cell. This is equivalent to imposing that one unit of the degrader chemical "reacts" with precisely one unit of the antibiotic. There might indeed be different stoichiometries in natural communities. Still, this condition allows us to make conclusions directly about the interaction graphs without any confounding effects due to the reactions' dynamics.

Next, we consider the birth and death of individuals: The growth rates of individuals of a given phenotype are decided using the method followed in the main text Methods section. Following Vetsigian [4], the effect of the antibiotics on the sensitive strains is decided by a dose-response curve with a threshold dosage below which antibiotics have no effect. Suppose the antibiotic concentration of any antibiotic (that A is sensitive to) is above the threshold. In that case, A is killed with some probability, a function of the antibiotic concentration. The dose-response curve gives the probability of dying as a function of external antibiotic concentration:

$$P_d(c) = \begin{cases} d & c < \tau \\ d + (1-d)(1-e^{k(\tau - c)}) & c \geq \tau \end{cases} \tag{A.3}$$

where the constants τ and k are to be determined. Respectively, they denote the threshold concentration value and the susceptibility of the sensitive strain i.e., how quickly the probability of death asymptotes to 1. These constants are determined by imposing two constraints on $P_d(c)$. Let $G(r)$ denote the value of the 2-dimensional Gaussian at a distance r from the peak (recall that it is spherically symmetric). Since u_p is the volume of antibiotic secreted by a producer every time step, $u_pG(r)$ is the concentration, post one application of the Gaussian filter, at a distance r. Then the conditions imposed are

$$P_{death}(u_pG(1)) = 0.99 \tag{A.4}$$

$$P_{death}(u_pG(\sigma)) = 0.9 \tag{A.5}$$

These constraints correspond to a “common sense” expectation from the dose-response curve. The first constraint is motivated by the expectation that the death probability must be very high (but perhaps not 1) for all neighbours of the producer i.e., all individuals at distance 1. The second corresponds to the expectation that at a distance of σ (here $\sigma > 1$), the death probability must be high, but lesser than the death probability at distance 1. Let

$$c^* = \frac{\ln(\frac{0.1}{1-d})}{\ln(\frac{0.01}{1-d})}.$$

Solving the two constraints for τ and k simultaneously, we have

$$\tau = \frac{u_p(c^*G(1) - G(\sigma))}{c^* - 1} \tag{A.6}$$

$$k = \frac{(c^* - 1)\ln(\frac{0.1}{1-d})}{u_p(G(1) - G(\sigma))} \tag{A.7}$$

The width σ of the Gaussian filter is set as $K_P/3$. This is because K_P in the mixed-inhibition zone model was interpreted as the area outside which the antibiotic does not have an effect. We assume the area of the Gaussian inside a distance of 3σ is an approximation of this area of effect since it contains 99.7% of the area under the normal distribution curve.

For the dispersal of offspring, we consider a dispersal neighbourhood $\mathfrak{D}$ consisting of some arrangement of grid cells neighbouring the cell that A occupies. If any of these cells are empty, then another individual belonging to the same strain as A is born with probability r, a function of the growth rate of A and the antibiotic concentration on that grid cell. Then this new individual is placed at a grid cell picked uniformly from the empty cells in $\mathfrak{D}$.

This simulation is then run for many generations, we then check for communities in which the strain abundances are nonzero for a long time. In particular, we study the 1-antibiotic PSD motif and simulate its dynamics over a large parameter range. To compare the dynamics under different parameter values after the system has relaxed to an attractor, if it exists, we take the average of the abundance over the last 500 generations, where the simulation was run for a total of 6000 generations. The implicit assumption we are making here is that the attractor will be reached after 6000 generations, and this generally seems to be true in our simulations.

A.3 Supplementary figures

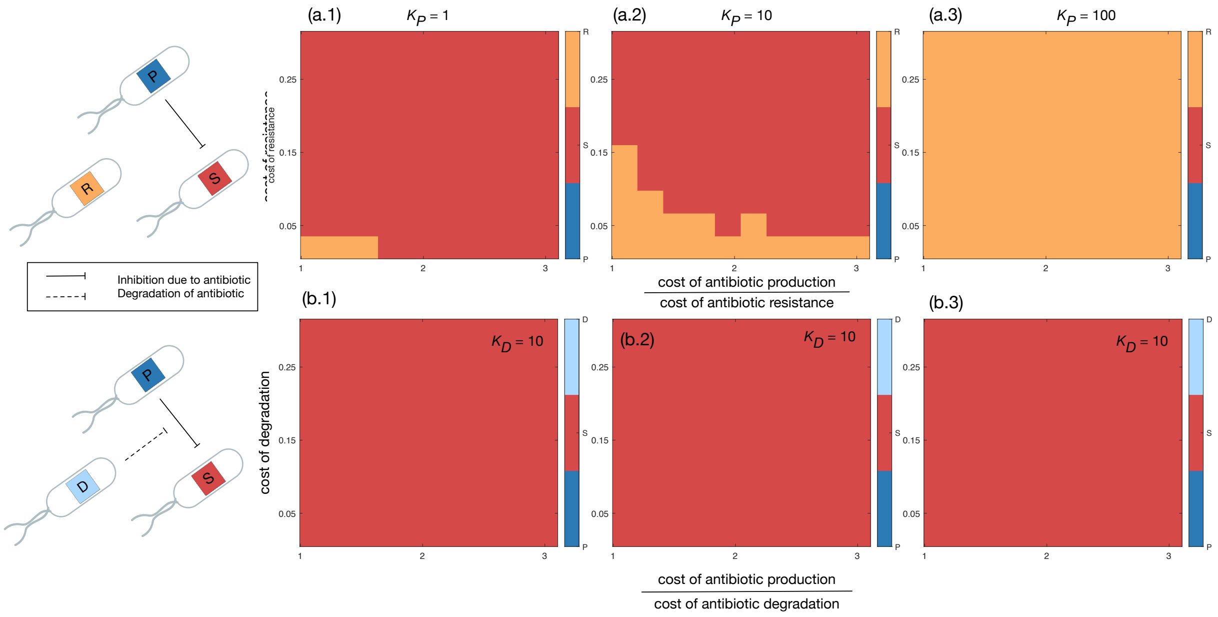

Fig. A.5: Interaction via one antibiotic. The communities [P,S,R] and [P,S,D] are studied across different parameter ranges, top and bottom row respectively. Neither community displays coexistence, and all trajectories lead to the vertex, i.e. one strain fixes and forms the whole population. In [P,S,R] (top row), it can be either the Sensitive (S) or Resistant (R) strain; at low K_P the sensitive strain wins, and at higher K_P, the degrader strain wins. In contrast, under the interaction graph [P,S,D], the sensitive strain (S) wins for all parameters checked.

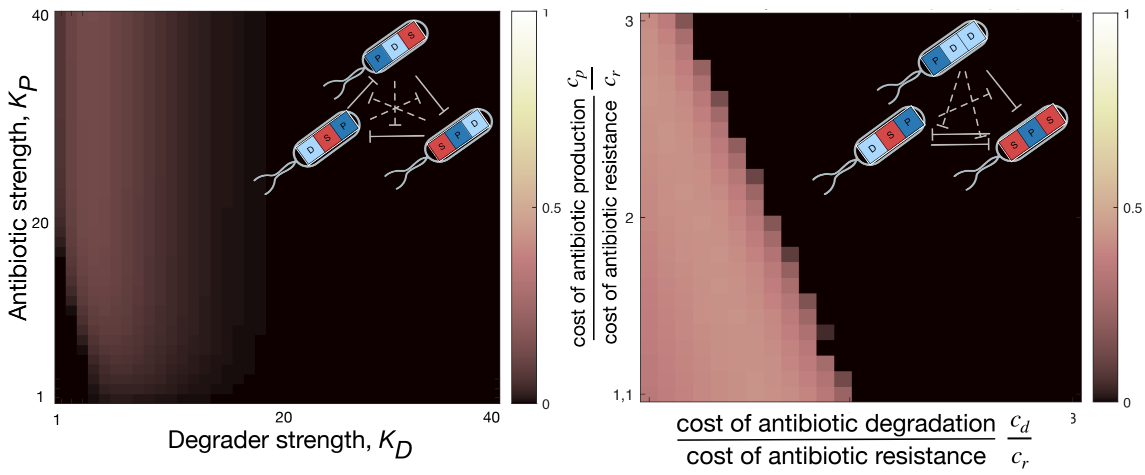

Fig. A.6: Robustness of the circulant vs. acirculant communities with 3 PSD motifs. The right panel is a reproduction of panel (c.1) of Figure 4 in the main text. It shows that the acirculant 3-PSD community has a fixed point for low enough c_p/c_r and c_d/c_r, and that the community has high robustness around these fixed points. Robustness is measured as 1 - the spectral radius of the appropriate Jacobian matrix evaluated at the fixed point. The left panel of this figure shows the robustness of the circulant community with 3 PSD motifs as a function of K_P and K_D, which for a given value of (K_P, K_D), is independent of c_r, c_p, c_d. Robustness is highest for high K_P and intermediate K_D, but it never goes higher than the values reached by the acirculant community on the right.

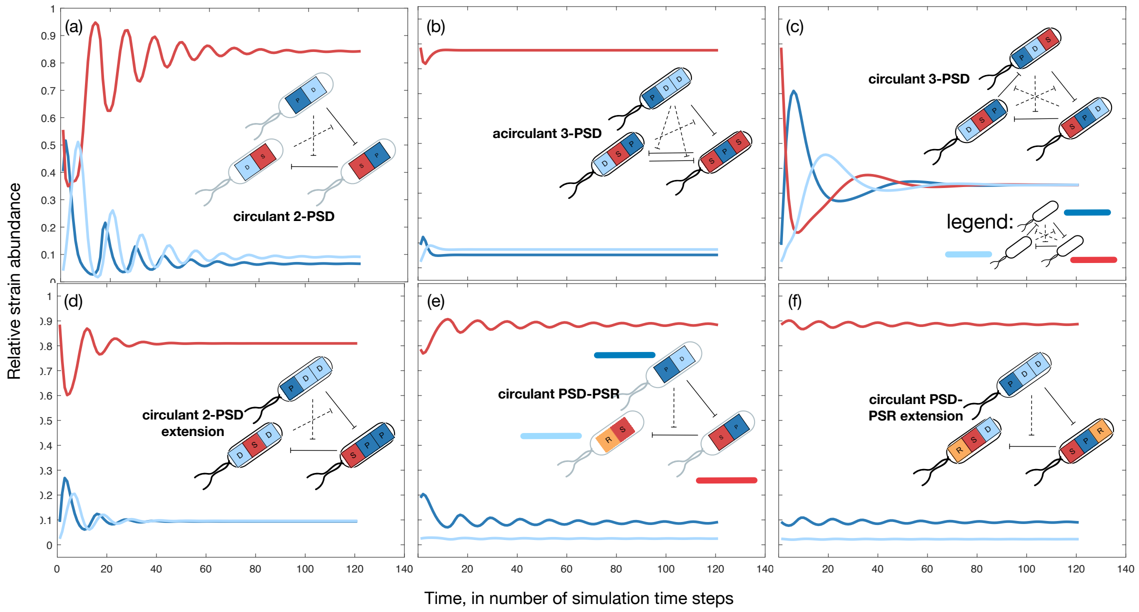

Fig. A.7: Trajectories leading to stable fixed points. Panels **(a,e)** present trajectories for the stably coexisting communities on two antibiotics. Panels **b,c,d,f** present trajectories for one example of the each category of stably coexisting community on three antibiotics. Initial conditions are randomly chosen when the basin of attraction is large; when the basin is smaller, the initial condition is chosen by perturbing slightly the value of the fixed point. Common parameters: $g = 1, c = 0.16, K_P = 40, K_D = 10$. In all panels except (e): $c_d = 1.4c_r, c_p = 2.5c_r$. Panel (e): $c_d = 2.2083c_r, c_p = 2.7625c_r$.

References

[1] I. M. Gessel and J. Li. Enumeration of point-determining graphs. *Journal of Combinatorial Theory, Series A*, 118(2):591–612, Feb. 2011.

[2] O. F. Inc. Entry a181230 in the on-line encyclopedia of integer sequences. *Entry A181230 in The On-Line Encyclopedia of Integer Sequences*, 2025.

[3] E. D. Kelsic, J. Zhao, K. Vetsigian, and R. Kishony. Counteraction of antibiotic production and degradation stabilizes microbial communities. *Nature*, 521:516–519, 2015.

[4] K. Vetsigian. Diverse modes of eco-evolutionary dynamics in communities of antibiotic-producing microorganisms. *Nature Ecology & Evolution*, 1(1), June 2017.
