# Supplementary material for: Coexistence rules for small, antagonistically interacting microbial communities
Source: PLoS Comput Biol. 2025 Dec 4;21(12):e1013763. doi: 10.1371/journal.pcbi.1013763 (PMC12697945; doi:10.1371/journal.pcbi.1013763)
Supplement: Attachment — Submitted filename: Review_of_PCOMPBIOL-D-24-02185.pdf [file pcbi.1013763.s003.pdf]

Review of PCOMPBIOL-D-24-02185 for PLOS Computational Biology

This study investigates the stability of microbial communities composed of a given small number of microbial strains (N) and subject to a given small number of antibiotics (M), which mediate the interactions between microbial strains. Each strain can either produce (P), degrade (D), resist (R), or be sensitive (S) to each antibiotic. For each combination of small N and M, the authors focus on finding the P-D-R-S motifs that provide long-lasting coexistence for all strains. Grounding first on a standard (deterministic) adjusted replicator dynamics model, they study the fixed points of community composition and their stability in well-mixed environments. Additionally, the authors develop and simulate a stochastic spatial model of these communities in a 2D hexagonal grid, including the spatial diffusion of the antibiotic and degrader units. Results show that a producer-sensitive-degrader motif is typically necessary for each antibiotic (but not always sufficient) for the stability of the microbial community, which is enhanced in spatial settings.

Overall, I found this study interesting, and, to the best of my knowledge, its results are novel. The subject is relevant because, on one hand, engineering more stable microbial communities (e.g. in bioreactors) can have a strong impact for society and, on the other hand, the general study of antimicrobial resistance can benefit public and animal health.

However, in my opinion, a few points must be addressed before the publication of this work. Below I detail a list of major and minor comments.

Major comments

1) The results of the mixed-inhibition zone model in Fig. 2c seem inconsistent with those of Table 1. According to Fig. 2c, no microbial community of N=4 strains and M=2 antibiotics presents stability. According to Table 1, however, the base community {PD,SP,DS} is stable when adding a single new strain with a phenotype of either PD, SP, DS, or DP. As I discuss in detail in major point 2) below, the previous first three new strains just repeat a pre-existing strain phenotype in the base community and should not be considered an extended version of the base community. However, critically, the last added strain, with phenotype DP, is a novel strain that provides a stable extended community of 4 different strains and 2 antibiotics: {PD,SP,DS,DP}. Hence, could the authors please clarify why the community {PD,SP,DS,DP} is stable in Table 1 but, if I understood correctly, Fig. 2c indicates that no community with 4 strains and 2

antibiotics is stable? If this is the case, please correct this and any other potential contradictions.

2) When studying extensions of stable communities, I consider that adding additional strains with the same antibiotic phenotypes as any pre-existing strain, or adding additional antibiotics with the same action as any pre-existing antibiotic, should not be considered as a different/extended community. To my understanding, the former might just increase the initial population of the repeated strain, and the latter might just double the strength of the repeated antibiotic. Could the authors please amend this thorough the manuscript (and in Table 1 in particular)?

Moreover, if one only allows non-repeated strain phenotypes and non-repeated antibiotics, it might be possible to calculate the number of distinct communities with N strains and M antibiotics. Namely, following the convention of the Supplementary Information section A.1, the different microbial communities with N strains and M antibiotics correspond to the set of NxM matrices, with entries in {1,2,3,4}, where all rows and columns are unique, and without row or column permutations. The number of NxM {1,2,3,4}-matrices with unique rows and columns can be derived similarly as in https://math.stackexchange.com/questions/3101130/the-number-of-m-times-n-binary-matrices-with-all-rows-distinct-and-all-column, which follows the entry A181230 of the OEIS https://oeis.org/A181230. However, note that one needs to replace the “2” in the formula (derived for binary matrices) by a “4” (for this papers’ ternary matrices), and the “m” and “n” notation might need to be exchanged. Then, to remove the redundancy due to row and column permutations, one needs to divide the result of the previous formula by the product of N factorial and M factorial, i.e., divide by (N!*M!).

This might be relevant for the results in Fig. 1c (and in the corresponding algorithm) once any repeated strains and/or antibiotics are removed from each community.

3) The paper would improve with more quantitative results for the non-spatial case. Currently, to my understanding, the non-spatial results are limited to the qualitative tables of Fig. 2c and Table 1 (which indicate the stability of the communities) and only two quantitative examples (those in Fig. 2b). I suggest that, for the stable case {PDS,SPD,DSP}, as well as for all the stable extended communities of Table 1, the authors provide similar plots as those in Fig. 2b (either in the main manuscript or, at least, in the supplementary information).

Regarding the stochastic spatial case, and if my understanding is correct, the stability of a community is assessed by averaging strain abundances in the last 500 generations (of the 6000 total simulated generations) and checking that no strain has zero average abundance in that window. If so, then microbial communities showing limit cycle oscillations are considered stable in the stochastic spatial model, as opposed to the non-spatial analysis. Moreover, the spatial model accounts for stochasticity whereas the non-spatial mixed-inhibition zone model does not. It would be useful if the authors con discuss in more detail how these differences should be taken into account when comparing the results of the spatial and non-spatial models. Do limit cycles account for a large fraction of the final states of the stable communities, either in the non-spatial or in the spatial case?

Minor comments

1) On one hand, the authors consider antibiotic degradation, enforced by degraders (D), which is indeed a common resistance mechanism that protects sensitive cells (S). On the other hand, they account for ``intrinsic'' resistance, which protects resistant cells (R) from a given antibiotic but does not protect S cells.

However, there are microbes that present other resistance mechanisms that could further inhibit S strains in the presence of antibiotic, such as efflux-pumps. These microbes pump out the antibiotic, further exposing S cells to even more antibiotic. The impact of this additional type of microbes on the results of this paper could be briefly discussed, e.g., in the Discussion section.

2) The authors consider that the fitness cost of degradation of antibiotic is larger than that of intrinsic resistance. Although perfectly reasonable, this does not always need to be the case. Would the resulting dynamics change significantly if D's fitness was larger than R's fitness?

3) Footnote 1 in page 6 seems to indicate that the authors assume equal metabolic costs for all antibiotics. Moreover, in the mixed-inhibition zone model, all of the N strains seem to always start with the same initial proportion of microbes (set at 1/N?). However, this is only hinted in the caption of Figure 2. If this is the case, both the equal metabolic cost across antibiotics and the equal starting fraction across strains, should be clearly stated in the main text when the mixed-inhibition zone model is introduced.

4) Please make explicitly clear in the corresponding captions that Figures 2-3 and Table 1 refer to the mixed-inhibition zone model (without spatial structure).

5) At the end of the caption of Fig. 3, please cite Table 1 explicitly when referring to “the table that records combinations of N and M that support stable coexistence states”.

6) Each antibiotic has a biocidal effect on the corresponding sensitive strains. Would the dynamics change significantly in the case of biostatic antibiotics, where only the growth rate of S cells is affected? It might not be the case for the replicator dynamics of the mixed-inhibition zone model, but would the dynamics change for the spatial stochastic model?

7) In the analysis of base community extensions: when adding a new strain, do the unstable extended communities typically return to their original, stable base community?

8) When introducing the stochastic spatial model for the first time, I would suggest mentioning explicitly that it runs on a 2D (hexagonal) grid.

9) In line 246, the authors mention a coexistence region in Fig. 5 in green, but there is no green in this figure.

Supplementary information (SI):

10) In the third line of the caption of figure A.1, the last "Sensitive" should be replaced by "Resistant".

11) As indicated in line 129 of SI, in the stochastic spatial simulations, at each time step authors ``iterate over all the grid cells in the lattice’’. However, if a microbe in the currently evaluated grid cell replicates, and its offspring spawns into the next grid cell to be evaluated, its offspring can replicate again in the

same time step, and so on. If I understood correctly, this might generate artifacts (although the final stability of the community is probably robust to this). Could authors please comment on this point?

12) As indicated in lines 136-138 of SI: degrader units from degrader cells are placed in neighbouring cells, whereas antibiotic units from antibiotic cells are placed in the focal cell. Why is so? Does not it enforce a bias in the diffusivities, with larger degrader diffusivity than antibiotic diffusivity at equal Kd and Kp?

13) Lines 138-140 of the SI might have to be reordered (e.g. line 138 ends with “diffused as above”, but diffusion is explained in lines 139-140).

14) In line 150 of the SI: authors might have to correct "in the previous section" as this is not explained in the SI but in the main manuscript.
