# Supplementary material for: Coexistence rules for small, antagonistically interacting microbial communities
Source: PLoS Comput Biol. 2025 Dec 4;21(12):e1013763. doi: 10.1371/journal.pcbi.1013763 (PMC12697945; doi:10.1371/journal.pcbi.1013763)
Supplement: Attachment — Submitted filename: Response to Reviewers.pdf [file pcbi.1013763.s004.pdf]

Notes on revision made to manuscript Paper #PCOMPBIOL-D-24-02185

The authors would like to thank the editor and reviewers for their constructive comments and suggestions; they have helped improve the quality of this manuscript. We also thank you for your patience – the comments prompted a thorough revision, including various amounts of re-analysis, extra analyses, and rewriting.

This more thorough analysis has led us to find new results, in light of which the manuscript needed a substantial revision. In particular, the PSD motif is not as necessary as previously inferred, and exactly how this is borne out is treated more carefully in the resubmitted version. The section on the mixed-inhibition zone (non-spatial) model, and associated Figures (2,3, new 4) have therefore changed. We also removed the "Extensions" subsection in favor of presenting the new results more clearly, as we agreed with the reviewers that the earlier analysis was overly diffuse. The writing has also changed in a few places to improve tone and make more precise statements, especially in the Results section, but also in the abstract.

For the reviewers' convenience, we highlight our responses in blue. To avoid confusion with prior line numbering, in the resubmitted version we cite locations using the abbreviation NL ("New Line") to refer to specific line numbers in the revised manuscript. We also provide a point-by-point response to each reviewer comment in the accompanying document and include diff.pdf, which highlights all changes to the manuscript relative to the previous submission.

We are grateful for the opportunity to revise and resubmit, and we hope the revised manuscript meets the journal's standards. We would welcome any further feedback.

Response to Additional Editor Comments

Reviewer Comment 0.1 — We have now received 3 reviews. These are largely positive, but some significant technical revisions are needed. Specifically:

Reviewer 1 highlights an apparent contradiction: Table 1 lists {PD, SP, DS, DP} as stable whereas Fig 2c shows no 4-strain/2-antibiotic stability; they also ask for quantitative trajectories for all "stable" graphs and a clearer treatment of limit cycles versus fixed points.

Reviewer 2 requests a formal definition of "cyclicity", explicit counting of non-redundant graphs, parameter ranges for the 900 (K_p, K_d) sets in Fig 4, and clearer rules governing extensions in Table 1 (why repeated phenotypes are excluded for strains but not for antibiotics).

Reviewer 3 endorses acceptance as-is.

Reply: Thank you for summarizing the reviews. We are encouraged by the overall positive assessments and have undertaken the significant technical revisions requested. We are grateful for the constructive feedback and believe the revisions substantially strengthen the manuscript. We welcome any further comments.

Essential Revisions

Reviewer Comment 0.2 — Resolve Fig 2c/Table 1 mismatch and state how redundancy filtering (identical rows/columns) is handled.

Provide a mathematical definition of cyclicity and prove (or numerically demonstrate) why PSD + cyclicity is sufficient but not PSD alone (e.g. {PP, SS, DD}).

Add quantitative time series (or phase-plane plots) for every graph deemed stable; note when stability is via limit cycles.

Document full parameter sweeps (c_p, c_d, c_r, K_p, K_d), initial conditions, and replicate strategy.

Improve figure/table legends, fix minor label/footnote errors, and harmonise metabolic-cost statements.

Please also provide point-by-point responses to all the Reviewers' comments.

Reply: Thank you for the clear and constructive suggestions. We have thus scrutinized the manuscript and incorporated all the changes suggested by the editor and the reviewers.

Journal Requirements

1. We ask that a manuscript source file is provided at Revision. Please upload your manuscript file as a `.doc`, `.docx`, `.rtf` or `.tex`. If you are providing a `.tex` file, please upload it under the item type "LaTeX Source File" and leave your `.pdf` version as the item type "Manuscript".
2. Please upload all main figures as separate Figure files in `.tif` or `.eps` format. For more information about how to convert and format your figure files please see our guidelines: `https://journals.plos.org/ploscompbiol/s/figures`

Reply: We have adhered to the specified journal requirements in our resubmission.

Response to the reviewers

Reviewer 1

Reviewer Comment 1.1 — The manuscript presents a graph-based theoretical model to explain biodiversity in microbial communities, focusing on how antibiotic production and degradation promote coexistence. The core claim is that the producer-sensitive-degrader (PSD) motif, as previously analyzed, is critical for enabling coexistence among microbes. By eliminating redundant (symmetric) topologies, the authors reduce the complexity of possible configurations and aim to identify exact rules that allow for stable coexistence in communities producing and degrading antibiotics. This approach is significant for the field because it seeks to formalize and generalize the principles underlying microbial diversity, potentially guiding experimental and synthetic community design.

Strengths

- The manuscript addresses an important question: what structural features in microbial interaction networks allow for stable coexistence despite antagonistic interactions.
- The use of graph theory to systematically explore the space of possible community interactions could clarify which network motifs are essential for stability.

Reply: We thank the reviewer for their assessment. We are pleased that the significance of our graph-based framework and its potential implications for understanding and engineering microbial communities were appreciated. We are very grateful for the constructive comments, and hope that our revisions satisfactorily address most concerns.

Reviewer Comment 1.2 — Areas for improvement
The manuscript lacks a clear and concise statement of the "exact rules" needed for stability. While the PSD motif's importance is discussed, the criteria for stability in more complex networks are not clearly articulated. For example:

- What fraction of antibiotics in the network must have a PSD motif?
- Must every configuration be cyclic, and what does "cyclic" mean in this context? Though examples of this cyclicity are presented I think a proper definition is needed, and from it to derive the "magnitude" of cyclicity needed as a rule.
- How do complexity and symmetry of configurations affect stability?

Reply: We now try to answer these questions, to the extent feasible, explicitly in our manuscript.

- Fraction: In light of our new results, it is possible for a community to coexist while not all of the antibiotics in the interaction graph have a PSD motif. But at least one PSD motif is necessary, and having more than one dramatically increases the size of the community's basin of attraction (see Figure 2). Fractions are mentioned explicitly on NL215 (NL = New Line), and NL250+.
- Cyclicity: We have defined cyclicity as the property of the strain-by-antibiotic interaction matrix being in a kind of "circulant" arrangement. Concretely, we call an interaction matrix as cyclic if the columns all contain identical (or similar, in the case of Degrader vs. Resistant) phenotypes, except that each column is shifted one row downward relative to the previous column. Notably, cyclicity in interactions is a property of how interactions along *multiple* antibiotics are arranged and it would not be well-defined if strains in a community interacted only via one antibiotic. In the two-antibiotic, three-phenotype setting this yields a unique fully cyclic community [PD,SP,DS], which we call the circulant community with two PSD motifs. The "magnitude of cyclicity" is the number of antibiotic columns that participate in this one-step rotational pattern under a single shift. Cyclicity is explicitly defined in the paragraph starting from NL195+.
- Complexity and symmetry: We now include a dedicated subsection on this topic (beginning at NL255; see the *Results* and new Fig. 4). Arranging PSD motifs across a greater number of antibiotics broadens the parameter range that supports a stable state, but it appears to reduce robustness to perturbations.

Reviewer Comment 1.3 — The methods and parameter choices are not sufficiently detailed, making it difficult to assess the robustness of the findings. For example:

- How is stability defined and quantified? For a given topology are eigenvalues searched on multiple parameter sets?

- What makes one configuration more stable than another? Is it just a matter of having a larger attractor, or still being stable in the face of extensions?
- What is the range of N and M being studied here? In the text both are not larger than 3 but in figure 2 it seems N can reach to 5. Are there any constraints such has M¡=N?
- What are the values of parameters scanned?

Reply:

- Stability: We numerically iterate our equations and search for fixed points. This fixed point is called stable if the Jacobian evaluated at the fixed point has a spectral radius less than 1 (this condition is the discrete-time analogue of the continuous-time criterion of having dominant eigenvalue less than 0). This is mentioned on NL134. When we search through the space of all communities, we compute this spectral radius at two parameter combinations, chosen by detailed analysis of specific communities of interest. The parameter values are detailed in NL156. During this detailed analysis (which we present in Figures 2 and 4), we compute the appropriate eigenvalues on a wide range of parameter values.
- There are a priori two answers to this question: the robustness to dynamical perturbations (by how much is the spectral radius smaller than 1?), and the size of the basin of attraction (how many initial conditions lead to the steady state in question?). We analyse both these measures for some communities in the resubmission (Figure 4). Stability in the face of extensions is no longer a consideration since we have chosen to exclude this section.
- We shifted panel (c) of the old Figure 2 to become a table on its own (new Table 1). This should clarify all the confusion around N, M values scanned.
- The parameter values are now mentioned more clearly at the beginning of the Results section (NL156), and also at the end of every figure legend.

Reviewer Comment 1.4 — Line 169- "for any parameter value- all strains have equal abundance at equilibrium" is a very strong statement and I'm not sure I understand its source.

Reply: This is a fair point, "for any parameter value" is not technically true. But it is true that the strains in the community in question [PDS, SPD, DPS] are all symmetric, in that they all have the same phenotype (just in a circulant manner), and so they incur the same metabolic cost if we assume that the costs for different antibiotics is the same. As a consequence (of this and the circulant interaction structure), whenever there exists a fixed point, the strains all have equal abundance. This fixed point, however, can cease to exist at high K_D and low K_P, see Fig. A6 in the SI.

Also relevant here is that we now assume, in the exhaustive search, that the antibiotics have slightly different metabolic costs (see NL108+). We implement this by adding a small, independently picked, random number to the cost of each antibiotic, just so we remove any interaction graphs that arise as stable only because of this unrealistic assumption. This would also give the strains in [PDS, SPD, DPS] slightly unequal abundance.

We have removed the delinquent sentence.

Reviewer Comment 1.5 —

- Table 1's purpose and message are somewhat unclear. While the manuscript text refers to it as holding a "complete record," the legend describes it as listing "notable extensions," which creates some confusion. Additionally, the table only presents stable extensions for strains, and unstable extensions for antibiotics and without clearly stating why. For greater clarity and completeness, I recommend the authors:

  1. clarify in both the text and the legend what Table 1 is intended to show,
  2. provide quantification or at least a summary of the number of possible extension of which how many are stable/unstable,
  3. consider whether these extensions follow distinct rules that could be discussed,
  4. clearly explain the importance of the bolded resilient to permutation entries.

- Finally, the information may be more effectively conveyed as a figure or schematic rather than a table, which could help readers grasp the relationships and extensions at a glance.

Reply: We agree with the reviewer that this Table was incomplete and not the best visualisation in the world. Due to the additional length of the new text and figures, we have chosen to exclude this section in favour of more fully understanding the conditions for coexistence.

Reviewer Comment 1.6 — Fig. 4: The legend states that "15 simulations [were] carried for each of 900 parameter sets, varying Kp and Kd values," but each subplot seems to show only a single value. It appears that the different lines reflect initial lattice configurations, with other parameters fixed. Please clarify the meaning of the 900 parameter sets and how Kp/Kd variation is represented.

Reply: We apologise for the confusion, these figure captions were badly worded. The number 900 is from an earlier version of this figure and is a typo, we have removed it. Before clarifying, we should say that the earlier Figs 4 and 5 are now Figs 5 and 6.

The figure containing the heatmap presents simulation outcomes for a wide range of K_D and K_P (old Fig 5 / New Fig 6), and the figure that is focal to this reviewer comment (old Fig 4 / new Fig 5) presents the full population dynamics for specific parameter combinations picked from the heatmap.

We now clarify this in the text and figure legends.

Reviewer Comment 1.7 — Fig. 4: Including a small schematic of the community, as in previous figures, would also aid reader understanding.

Reply: Done, in both figures relating to the spatial simulations (they are now figs. 5 and 6 instead of 4 and 5, because of the new figure 4 arising from the non-spatial setting).

Reviewer Comment 1.8 — Regarding the extension section: when a new antibiotic is introduced that doesn't encompass a producer-sensitive the community stays stable. I wonder if this is true also in the more realistic case where the parameters are not equal to all antibiotics, or if there are any "rules" where this is true in this scenario. In other words- how robust is the community to such additions?

Reply: As mentioned at the beginning of this document, we have chosen to exclude the section on Extensions in the resubmitted version.

But still, the present results offer some insights on this question: it isn't true that all extensions that don't encompass a producer-sensitive are automatically stable, because these extensions still alter the growth rates of the strains (because of the additional metabolic costs). It would likely also change when these costs are not equal across the antibiotics. The relevant rule is probably only that an extension is stable if it does not disrupt the metabolic costs by "too much". We see support for this claim, since not all P,D,R-containing extensions of the circulant 2-PSD community are stable when we search $M = 3$. However, this is outside the scope of our work so we do not analyse this in much greater detail.

Minor comments:

Reviewer Comment 1.9 — Fig A.1 legend: "P, S, D, R where the letters stand for Producer, Sensitive, Degrader, and Sensitive respectively". Change the second Sensitive to Resistant.

Reply: changed.

Reviewer Comment 1.10 — Supplementary row 47 Fig A.2 – I would state that matrix 2 & 3 are indeed identical even if identities are important.

Reply: sentence rephrased.

Reviewer Comment 1.11 — Clarify: in the methods line 437 it is said that: "the costs for each antibiotic are the same", while in the footnote for line 148 it is said that the assumption of identical metabolic costs is made for convenience but is omitted when stable fixed point disappeared when the two antibiotics have different metabolic costs.

Reply: We have clarified this point in the main text. To remove any such edge cases where the community appears stable only when the antibiotics have exactly identical metabolic costs (a scenario that is extremely unlikely), we changed our models to assume near-identical, but not exactly identical, metabolic costs. We implement this by fixing a cost of resistance c_r, and adding a small random number to it for each antibiotic. See main text for more details, NL108+.

The footnote in question has been removed.

Reviewer Comment 1.12 — Clarify: In line 146 it is said that one of 430 distinct communities had stable internal fixed point, but it is not clear if these are different runs of the $N = 3$, $M = 2$ with different parameters? Multiple topologies?

Reply: We meant different topologies, but this part of the results section has changed enough that the sentence that this comment pertains to does not exist anymore. And moreover, we now know that the result in the focal sentence of the older version is not true.

Reviewer Comment 1.13 — Add to the discussion some acknowledgment regarding synergistic interactions between antibiotics that are not as straightforward as the one described in the model (using the binomial formulation).

Reply: Thanks for the helpful comment. We have added a note (NL454+) in the Discussion section acknowledging that real-world antibiotic interactions can involve synergy beyond the independent-action assumption used in our model, NL457.

Reviewer Comment 1.14 — Add to the discussion a sentence regarding the costs of the different functions (production > Degrader etc.). In real microbial interactions the costs of production can be much lower, and condition dependent – for instance if the production only occurs when the density of the species is high enough or it is surrounded by other competing species.

Reply: We have added a line to the Discussion section to reflect that in natural systems, costs associated with production, degradation, or resistance may vary contextually and be regulated based on density or environmental cues, which is not modeled in our current framework, NL464.

Reviewer Comment 1.15 — Line 93: what is the definition of a "critical interaction graph"?

Reply: Thank you for commenting on this, in hindsight it is ambiguous usage of the word "critical". Here we mean that we explore the effect of spatial structure on the P-S-D motif interactions by focusing on a community with an *important* interaction graph. In particular, we focus on the PSD motif on one antibiotic, a community that does not exhibit a stable fixed point in a well-mixed scenario. We have removed this word, and replaced the sentence with "focusing on a community which does not exhibit a stable fixed point in a well-mixed scenario", NL92-93.

Reviewer Comment 1.16 — Figure 1: I think the example from Kelsic et al. there is a bit too symmetrical and another example (such as in Figure 2) should be added.

Reply: We have added an additional, less symmetrical example to Figure 1 for illustrative contrast.

Reviewer Comment 1.17 — Figure 1: Panel C is not very informative for the model understanding, but more for the computational reduction. Consider showing it elsewhere.

Reply: We agree that panel C of the old Figure 1 was not very informative. Additionally, on the recommendation of another reviewer, we also have an exact expression for how many distinct interaction graphs exist. We have therefore removed that panel entirely, and replaced it by a simple schematic showing the impact of degradation as a resistance mechanism.

Reviewer Comment 1.18 — Fig. 1 legend: "linked to equations in section" – no section is detailed.

Reply: corrected.

Reviewer Comment 1.19 — The sentence in lines 312–313 should be rephrased. It is clearly not sufficient to have PSD motifs to every antibiotic.

Reply: Thank the reviewer for pointing this out. This sentence has been replaced throughout by more precise statements in terms of the number of PSD motifs and the circulant nature of interaction matrices.

Reviewer Comment 1.20 — A better explanation of the meaning of "these PSD motifs must balance each other" (line 313) would be appropriate – if I understand correctly this is what the authors mean by "cyclicity"?

Reply: Yes, the reviewer is correct. We have now defined this a bit more exactly in the results section, see NL196.

Reviewer 2

Reviewer Comment 2.1 — The work by Athreya et al. investigates the complex interplay of antibiotic production, resistance, and degradation in shaping microbial diversity using a theoretical framework. The core of the manuscript focuses on identifying specific interaction patterns, or "motifs," that are critical for microbial coexistence in interaction graphs, specifically a "producer-sensitive-degrader" (PSD) motif. The study provides mechanistic insights by establishing exact rules for coexistence in small microbial communities and demonstrates that the PSD motif, particularly when combined with a cyclic interaction structure, is sufficient for stable coexistence in well-mixed populations. The authors also use individual-based simulations to explore the role of this motif in spatially structured populations, finding that community coexistence remains robust across a wide range of antibiotic and degrader diffusivities. The findings emphasize that antagonism, often perceived as a barrier to biodiversity, can drive diversity under specific interaction patterns. I really like how the manuscript was structured overall - all the sections are drafted well - and enjoyed reading it. I do not have any comments and am happy for this paper to be accepted.

Reply: Thank you for your time and encouraging comments!

Reviewer 3

Reviewer Comment 3.1 — This study investigates the stability of microbial communities composed of a given small number of microbial strains (N) and subject to a given small number of antibiotics (M), which mediate the interactions between microbial strains. Each strain can either produce (P), degrade (D), resist (R), or be sensitive (S) to each antibiotic. For each combination of small N and M, the authors focus on finding the P-D-R-S motifs that provide long-lasting coexistence for all strains. Grounding first on a standard (deterministic) adjusted replicator dynamics model, they study the fixed points of community composition and their stability in well-mixed environments. Additionally, the authors develop and simulate a stochastic spatial model of these communities in a 2D hexagonal grid, including the spatial diffusion of the antibiotic and degrader units. Results show that a producer-sensitive-degrader motif is typically necessary for each antibiotic (but not always sufficient) for the stability of the microbial community, which is enhanced in spatial settings. Overall, I found this study interesting, and, to the best of my knowledge, its results are novel. The subject is relevant because, on one hand, engineering more stable microbial communities (e.g. in bioreactors) can have a strong impact for society and, on the other hand, the general study of antimicrobial resistance can benefit public and animal health. However, in my opinion, a few points must be addressed before the publication of this work. Below I detail a list of major and minor comments.

Reply: We thank the reviewer for their assessment, and for engaging deeply with our work. We are very grateful for the constructive comments, and hope that our revisions satisfactorily address most concerns.

Reviewer Comment 3.2 — 1) The results of the mixed-inhibition zone model in Fig. 2c seem inconsistent with those of Table 1. According to Fig. 2c, no microbial community of N=4

strains and M=2 antibiotics presents stability. According to Table 1, however, the base community {PD,SP,DS} is stable when adding a single new strain with a phenotype of either PD, SP, DS, or DP. As I discuss in detail in major point 2) below, the previous first three new strains just repeat a pre-existing strain phenotype in the base community and should not be considered an extended version of the base community. However, critically, the last added strain, with phenotype DP, is a novel strain that provides a stable extended community of 4 different strains and 2 antibiotics: {PD,SP,DS,DP}. Hence, could the authors please clarify why the community {PD,SP,DS,DP} is stable in Table 1 but, if I understood correctly, Fig. 2c indicates that no community with 4 strains and 2 antibiotics is stable? If this is the case, please correct this and any other potential contradictions.

Reply: Thank you, this contradiction was a consequence of not enough starting points provided for the equation solver, leading to the simulation finding the fixed point in one scenario and not the other. {PD,SP,DS,DP} does indeed exhibit a stable steady state. However, this result was an artefact of assuming that the two antibiotics have identical metabolic costs of production/degradation/resistance associated to them (c_p, c_d, c_r respectively). We remedy this by assuming now that the antibiotics have different (but still similar) metabolic costs when doing the exhaustive search through interaction graphs (detailed in the main text, NL108+, NL="New Line"). This is implemented as follows: in our analyses, we fix c_r and then vary c_p/c_r and c_d/c_r. We choose a scale for c_r (say $c_r^{(0)}$) and choose the costs of intrinsic resistance against the two antibiotics as $c_r = c_r^{(0)} + 0.0005 * \text{Uniform}[-1,1]$, where $\text{Uniform}[-1,1]$ is a random number chosen uniformly from the interval $[-1,1]$.

When accounting for this, the $N = 4, M = 2$ community in question ceases to exhibit a stable steady state (see Table 1 in the resubmitted version).

We have also increased 2-fold the number of initial conditions that the equations are solved from.

Reviewer Comment 3.3 — 2) When studying extensions of stable communities, I consider that adding additional strains with the same antibiotic phenotypes as any pre-existing strain, or adding additional antibiotics with the same action as any pre-existing antibiotic, should not be considered as a different/extended community. To my understanding, the former might just increase the initial population of the repeated strain, and the latter might just double the strength of the repeated antibiotic. Could the authors please amend this throughout the manuscript (and in Table 1 in particular)?

Moreover, if one only allows non-repeated strain phenotypes and non-repeated antibiotics, it might be possible to calculate the number of distinct communities with N strains and M antibiotics. Namely, following the convention of the Supplementary Information section A.1, the different microbial communities with N strains and M antibiotics correspond to the set of $N \times M$ matrices, with entries in $\{1,2,3,4\}$, where all rows and columns are unique, and without row or column permutations. The number of $N \times M$ $\{1,2,3,4\}$-matrices with unique rows and columns can be derived similarly as in `https://math.stackexchange.com/questions/3101130/the-number-of-m-times-n-binary-matrices-with-all-rows-distinct-and-all-column`, which follows the entry A181230 of the OEIS `https://oeis.org/A181230`. However, note that one needs to replace the "2" in the formula (derived for binary matrices) by a "4" (for this paper's quaternary matrices), and the "m" and "n" notation might need to be exchanged. Then, to remove the redundancy due to row and column permutations, one needs to divide the result of the previous formula by the product of $N!$ and $M!$, i.e., divide by $(N! \cdot M!)$.

This might be relevant for the results in Fig. 1c (and in the corresponding algorithm) once any repeated strains and/or antibiotics are removed from each community.

Reply: As mentioned at the beginning of this document, we have now chosen to exclude the section on Extensions in favour of the additional results that we now present. But yes, we agree that adding additional strains or antibiotics with the same phenotype/action only introduces redundancy and does not really constitute a new community. We have therefore redone all analyses that use the mixed-inhibition zone model with the additional step of a priori removing communities with repeated rows/columns.

Regarding the number of distinct communities: thank you very much, this is a good observation! We now include a reference to this calculation in the main text. However, we also acknowledge that this OEIS sequence provides guiding knowledge only on how many non-redundant, non-repeating communities exist, and does not constitute an algorithm to actually *enumerate* these non-redundant, non-repeating communities (which is what we need). Nevertheless, we are grateful for the reviewer for pointing us to this.

Reviewer Comment 3.4 — 3) The paper would improve with more quantitative results for the non-spatial case. Currently, to my understanding, the non-spatial results are limited to the qualitative tables of Fig. 2c and Table 1 (which indicate the stability of the communities) and only two quantitative examples (those in Fig. 2b). I suggest that, for the stable case PDS,SPD,DSP, as well as for all the stable extended communities of Table 1, the authors provide similar plots as those in Fig. 2b (either in the main manuscript or, at least, in the supplementary information). Regarding the stochastic spatial case, and if my understanding is correct, the stability of a community is assessed by averaging strain abundances in the last 500 generations (of the 6000 total simulated generations) and checking that no strain has zero average abundance in that window. If so, then microbial communities showing limit cycle oscillations are considered stable in the stochastic spatial model, as opposed to the non-spatial analysis. Moreover, the spatial model accounts for stochasticity whereas the non-spatial mixed- inhibition zone model does not. It would be useful if the authors can discuss in more detail how these differences should be taken into account when comparing the results of the spatial and non-spatial models. Do limit cycles account for a large fraction of the final states of the stable communities, either in the non-spatial or in the spatial case?

Reply:

- **Population dynamics trajectories:** We now provide example trajectories for all categories of stable communities in SI Fig. A7. We also now provide a Shiny app (works for any 3-strain community) that displays dynamics and the flow field on the simplex.
- **Limit cycles in the non-spatial case:** First the non-spatial model. In the non-spatial analysis of the 1-antibiotic PSD motif [P,S,D], there are never limit cycles, and the system always escapes to a vertex of the simplex, i.e. only one strain fixes and others go extinct (see Fig. A5 in the SI). So this is what the spatial case must be compared to, since this is the only community which we simulate on the lattice.

  Nevertheless, we also checked for limit cycles in the 2-antibiotic circulant PSD community (by asking if the trajectory periodically returns very close to the same point in the same order), and found that some trajectories exhibited limit-cycle-like behaviour (blue in Figure 1a, present document). But when we check these points' trajectories, the cycles are decreasing in amplitude (b) and eventually reach a fixed point (c). To make sure this is really a fixed point and not a stable

limit cycle with small amplitude, we zoomed in to the trajectories of only the second and third species; this confirms that it is only a fixed point (d). The oscillations just decrease in amplitude extremely slowly, which is why our method identifies them as limit cycles. They also happen at the edge of the regions between which stable fixed points cease to exist. We would therefore guess that there are likely no true limit cycles in the the 2-antibiotic circulant PSD community; and if there are, they do not occur very frequently in parameter space.

- **Limit cycles in the spatial case**: This scenario is a bit more inconclusive. However, we very (very) tentatively claim that there are no limit cycles in the lattice dynamics of the 1-antibiotic PSD motif, and the fluctuation is instead due to "quasi-cycles" [1], i.e. oscillatory trajectories to a fixed point (aka stable focus) which are constantly kicked away from the fixed point due to finite-size stochasticity. They look like limit cycles because they can never actually remain at the fixed point. We evaluated trajectories at each parameter combination by calculating (i) the discrete Fourier transform of the time series of population abundances, (ii) the autocorrelation of the time series of strain relative abundances. A trajectory exhibits a limit cycle if (i) the Fourier transform has a peak at intermediate x-coordinate, i.e. frequency, (ii) the autocorrelation function is itself oscillatory. A quasi-cycle would turn up as an oscillating decrease to zero in the autocorrelation (see Fig. 3 of [1]).

  We were unable to find a succinct metric that allows us to differentiate between a quasi-cycle and limit cycle over a large parameter space. To superficially get at which trajectories exhibit the most fluctuation, we computed the average coefficient of variation in the strains' trajectories in the last 50 sliding windows of length 100 timesteps each. This leads to Figure 2a (there are changes because we had to rerun the trajectories), which shows that the highest variation is for jointly high values of K_P and K_D. We then manually checked the autocorrelation for parameter combinations placed far apart from each other. This is shown in 2b-e, all of these and the other ones we have checked display the symptoms of either a stable node or a quasi-cycle as shown in [1].

  However, this analysis is more informal and less systematic, we chose not to include it in the resubmission; we provide it here for the reviewer's information.

Reviewer Comment 3.5 — Minor comment 1: On one hand, the authors consider antibiotic degradation, enforced by degraders (D), which is indeed a common resistance mechanism that protects sensitive cells (S). On the other hand, they account for "intrinsic" resistance, which protects resistant cells (R) from a given antibiotic but does not protect S cells. However, there are microbes that present other resistance mechanisms that could further inhibit S strains in the presence of antibiotic, such as efflux-pumps. These microbes pump out the antibiotic, further exposing S cells to even more antibiotic. The impact of this additional type of microbes on the results of this paper could be briefly discussed, e.g., in the Discussion section.

Reply: Thanks for the suggestion. We have now highlighted this assumption of our model and added a brief discussion on efflux-pump-mediated resistance in NL458 of the Discussion section.

Reviewer Comment 3.6 — Minor comment 2: The authors consider that the fitness cost of degradation of antibiotic is larger than that of intrinsic resistance. Although perfectly reasonable,

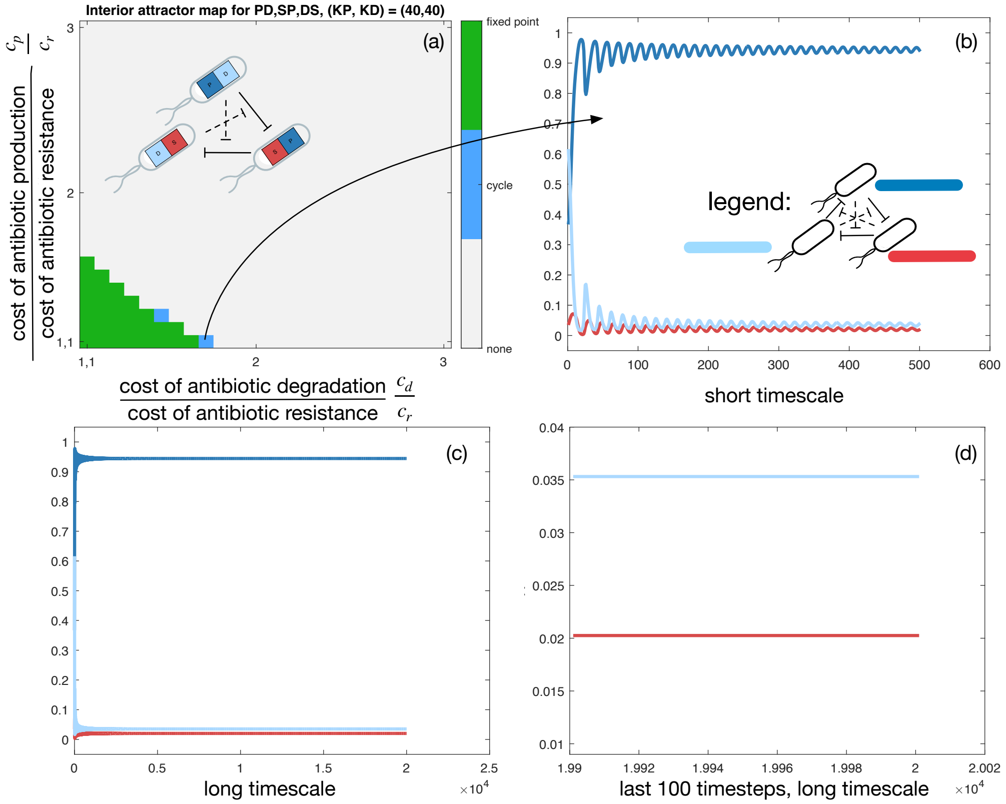

Figure 1: Parameters: $g = 1, K_P = 40, K_D = 40, c_r = 0.16, c_d = 1.733c_r, c_p = 1.1c_r$.

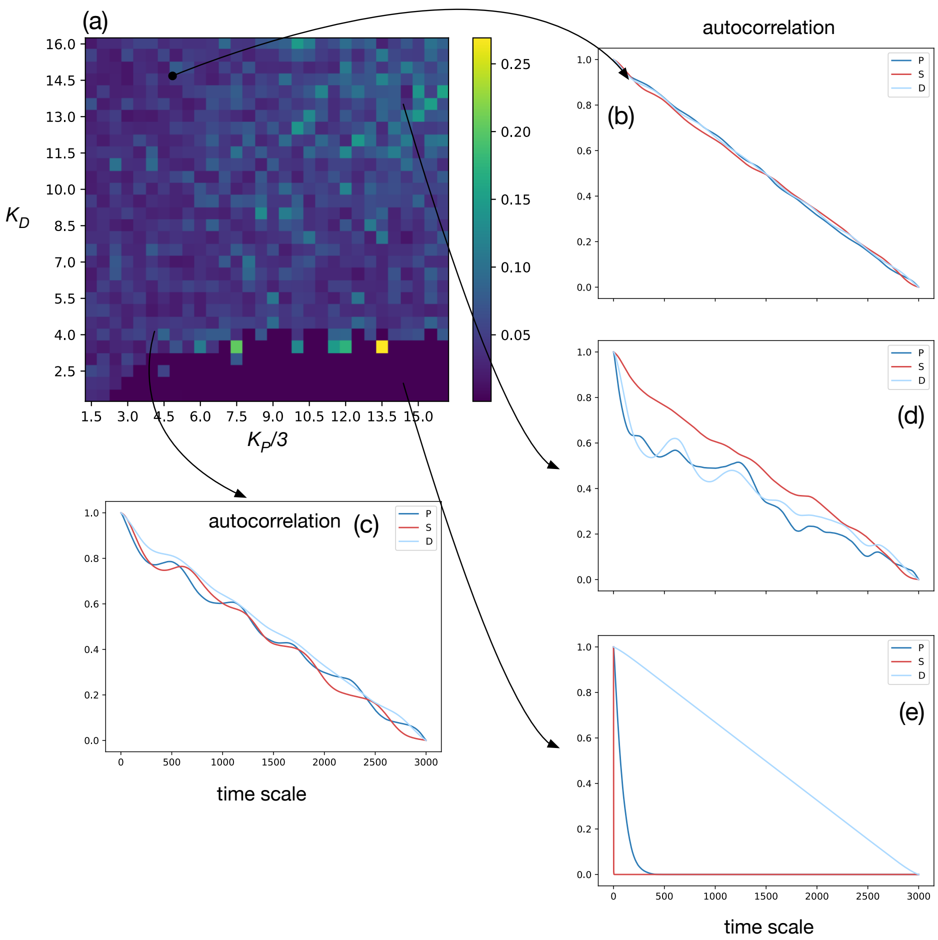

Figure 2: Parameters: $b = 0.7, d = 0.3, c_r = 0.16, c_d = 2.1c_r, c_p = 3c_r$, K_P and K_D as indicated.

this does not always need to be the case. Would the resulting dynamics change significantly if D's fitness was larger than R's fitness?

Reply: Breaking this assumption would affect the relative growth rates of strains exhibiting D or R phenotypes. To answer this question, we therefore analysed two communities that contain both D and R phenotypes and displayed/did not display stability in interesting ways: [PD,SP,RS] and [PR,SP,DS] (the former supports coexistence under our main-text assumptions, the latter not). In both communities, allowing for c_d/c_r and also c_p/c_r to be less than one did not change the regions of coexistence. Heatmaps in Figure 3.

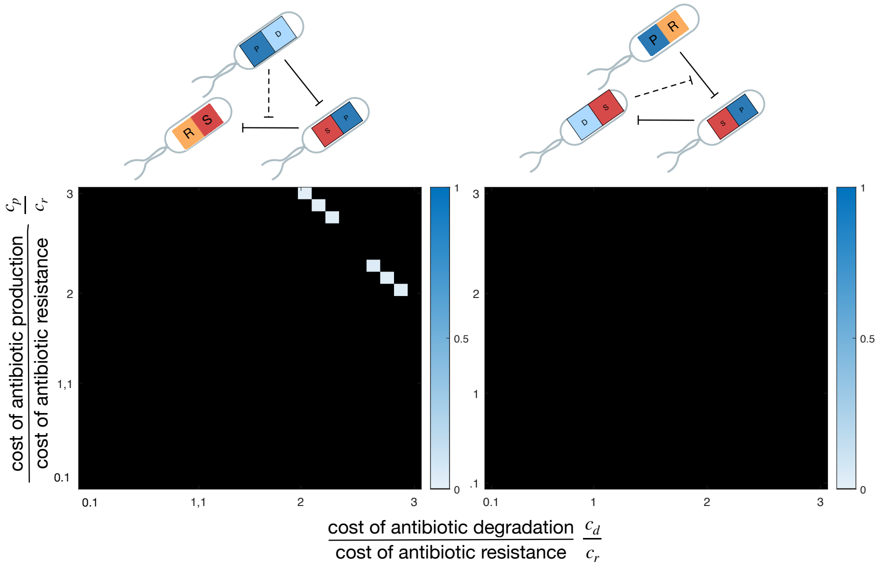

Figure 3: Parameters: $g = 1, K_P = 40, K_D = 10, c_r = 0.16$. c_d/c_r and c_p/c_r as indicated.

Reviewer Comment 3.7 — Minor comment 3: Footnote 1 on page 6 seems to indicate that the authors assume equal metabolic costs for all antibiotics. Moreover, in the mixed-inhibition zone model, all of the N strains seem to always start with the same initial proportion of microbes (set at $1/N$?). However, this is only hinted at in the caption of Figure 2. If this is the case, both the equal metabolic cost across antibiotics and the equal starting fraction across strains should be clearly stated in the main text when the mixed-inhibition zone model is introduced.

Reply: The assumption of equal metabolic costs across antibiotics has been altered, see NL108+ for the current assumptions.

Regarding the trajectories now presented in Fig. A7 of the SI: initial proportions are chosen randomly for communities which have large basins of attraction; when the basin is smaller, the initial condition is chosen by perturbing slightly the value of the fixed point. This is done to focus more on the nature of the approach to equilibrium (straight vs. oscillatory), and less on the exact values that the trajectories start from.

Reviewer Comment 3.8 — Minor comment 4: Please make explicitly clear in the corresponding captions that Figures 2-3 and Table 1 refer to the mixed-inhibition zone model (without spatial structure).

Reply: Done.

Reviewer Comment 3.9 — Minor comment 5: At the end of the caption of Fig. 3, please cite Table 1 explicitly when referring to "the table that records combinations of N and M that support stable coexistence states".

Reply: This table has been removed due to our exclusion of the section on Extensions.

Reviewer Comment 3.10 — Minor comment 6: Each antibiotic has a biocidal effect on the corresponding sensitive strains. Would the dynamics change significantly in the case of biostatic antibiotics, where only the growth rate of S cells is affected? It might not be the case for the replicator dynamics of the mixed-inhibition zone model, but would the dynamics change for the spatial stochastic model?

Reply: The reviewer is correct in noting that decreasing birth rate (say b) and increasing death rate (say d) are not in general identical. In infinitely large well-mixed populations, they are identical because only the difference $b - d$ is what matters. However, this changes when one has finitely many individuals. When spatial structure exists and population size is held constant, the picture is quite messy, see [2]. When there is no spatial structure but the population size is no longer constrained by the artificial assumption of constant size, the picture is again different, but quite clear: higher turnover, i.e. $b + d$ is selected against [3–6]. Now consider that a bactericidal treatment, by increasing death rate, increases turnover rates; a bacteriostatic treatment, by decreasing birth rate, decreases turnover rate. Therefore a bactericidal treatment should become more effective at low population sizes, meaning our results form the scenario where the antibiotics act the strongest – and we still see coexistence.

Reviewer Comment 3.11 — Minor comment 7: In the analysis of base community extensions: when adding a new strain, do the unstable extended communities typically return to their original, stable base community?

Reply: This is a good question, and the answer is usually yes. Of course, we have excluded this section now so we have not performed exact analyses to check if there are systematic changes in the size of the basin of attraction (there almost definitely are), but in all the cases we have checked, the newly added strain usually goes extinct and the composition settles onto the fixed point of the stable base community. There are probably parts of the initial condition space where this is not true (for example, if the population starts with an extremely large fraction of only the new strain).

Reviewer Comment 3.12 — Minor comment 8: When introducing the stochastic spatial model for the first time, I would suggest mentioning explicitly that it runs on a 2D (hexagonal) grid.

Reply: Agreed, now mentioned on NL 308.

Reviewer Comment 3.13 — Minor comment 9: In line 246, the authors mention a coexistence region in Fig. 5 in green, but there is no green in this figure.

Reply: We thank the reviewer for noting this discrepancy. We have now changed it to "mix shades of blue and red".

Reviewer Comment 3.14 — Supplementary comment 10: In the third line of the caption of figure A.1, the last "Sensitive" should be replaced by "Resistant".

Reply: Corrected.

Reviewer Comment 3.15 — Supplementary comment 11: As indicated in line 129 of SI, in the stochastic spatial simulations, at each time step authors "iterate over all the grid cells in the lattice". However, if a microbe in the currently evaluated grid cell replicates, and its offspring spawns into the next grid cell to be evaluated, its offspring can replicate again in the same time step, and so on. If I understood correctly, this might generate artifacts (although the final stability of the community is probably robust to this). Could authors please comment on this point?

Reply: Thank you for pointing this out. This effect does not actually take place, the writing in the Supplementary appendix was just suboptimal. Grid cells are updated "asynchronously" i.e. in a random order (see code file 'SimulationCode/spatial_model/eco-dyn.py', line 387-390 in Github repo), so the effect mentioned by the reviewer above is vanishingly unlikely. The relevant sentence in the Supplementary appendix has been clarified.

Reviewer Comment 3.16 — Supplementary comment 12: As indicated in lines 136-138 of SI: degrader units from degrader cells are placed in neighbouring cells, whereas antibiotic units from antibiotic cells are placed in the focal cell. Why is so? Does not it enforce a bias in the diffusivities, with larger degrader diffusivity than antibiotic diffusivity at equal Kd and Kp?

Reply: This is also an oversight, the antibiotic and degrader molecules are treated identically in all scenarios. Both molecules are placed at the focal cell (not the neighbouring cells) where the producer/degrader is living, and they diffuse according to the Gaussian filter parametrised by the diffusivities K_P and K_D.

Reviewer Comment 3.17 — Supplementary comment 13: Lines 138-140 of the SI might have to be reordered (e.g. line 138 ends with "diffused as above", but diffusion is explained in lines 139-140).

Reply: Done.

Reviewer Comment 3.18 — Supplementary comment 14: In line 150 of the SI: authors might have to correct "in the previous section" as this is not explained in the SI but in the main manuscript.

Reply: Corrected.

References

[1] Mario Pineda-Krch, Hendrik J. Blok, Ulf Dieckmann, and Michael Doebeli. A tale of two cycles – distinguishing quasi-cycles and limit cycles in finite predator–prey populations. *Oikos*, 116(1):53–64, October 2006.

[2] Sedigheh Yagoobi, Nikhil Sharma, and Arne Traulsen. Categorizing update mechanisms for graph-structured metapopulations. *Journal of The Royal Society Interface*, 20(200), March 2023.

[3] George W. A. Constable, Tim Rogers, Alan J. McKane, and Corina E. Tarnita. Demographic noise can reverse the direction of deterministic selection. *Proceedings of the National Academy of Sciences*, 113(32), July 2016.

[4] Michael Raatz and Arne Traulsen. Promoting extinction or minimizing growth? the impact of treatment on trait trajectories in evolving populations. *Evolution*, 77(6):1408–1421, March 2023.

[5] Ananda Shikhara Bhat and Vishwesha Guttal. Eco-evolutionary dynamics for finite populations and the noise-induced reversal of selection. *The American Naturalist*, 205(1):1–19, January 2025.

[6] Teemu Kuosmanen, Simo Särkkä, and Ville Mustonen. Turnover shapes evolution of birth and death rates. *Evolution*, May 2025.
