# Supplementary material for: Coexistence rules for small, antagonistically interacting microbial communities
Source: PLoS Comput Biol. 2025 Dec 4;21(12):e1013763. doi: 10.1371/journal.pcbi.1013763 (PMC12697945; doi:10.1371/journal.pcbi.1013763)
Supplement: Attachment — Submitted filename: Second_Review_of_PCOMPBIOL-D-24-02185_R1.pdf [file pcbi.1013763.s005.pdf]

Second review of PCOMPBIOL-D-24-02185_R1 for PLOS Computational Biology (Reviewer 3)

I would like to thank the authors for carefully (and satisfactorily) addressing all my comments. The authors have significantly improved the manuscript, and I am happy to recommend the publication of this work.

Some additional minor comments for this last version of the manuscript:

-The community on the right of Fig. 1 is denoted by "[PDS, DSP, SPD]" on line 169. Although the strain order is ultimately arbitrary, Fig 1b denotes it by [PDS, SPD, DSP], and it might be better to do so also on line 169 for clarity/consistency.

-On line 11 of the caption of Fig. 2 it is stated that "Costs and strengths are here assumed to be identical across antibiotics". Then, lines 14-15 repeat that: "Metabolic costs are assumed equal across antibiotics". Please merge or remove one of the statements.

-On lines 3-6 of the caption of Fig. 3, please explicitly indicate the corresponding panel letters (c), (b), (a), or (d) when enumerating the 4 categories of this set of communities (i), (ii), (iii), and (iv).

-In Fig. 5, the "(K_D, K_P) = (1.3, 13.9)" set of parameter values indicated in top-left and bottom-left panels are identical. Is this a mistake? Please check and correct if so.

-In the paragraph starting on line 337, it could be indicated that references to "(red)" and "(blue)" correspond to the heatmap in Fig. 6.

-On line 385, it might be necessary to add "number" right after "arbitrary". Please check.

Sincerely,

Lluís Hernández-Navarro
