# Supplementary material for: Coexistence rules for small, antagonistically interacting microbial communities
Source: PLoS Comput Biol. 2025 Dec 4;21(12):e1013763. doi: 10.1371/journal.pcbi.1013763 (PMC12697945; doi:10.1371/journal.pcbi.1013763)
Supplement: Attachment — Submitted filename: Review PlosComp.docx [file pcbi.1013763.s002.docx]

Antibiotic-mediated interactions underlying microbial diversity - review

The manuscript presents a graph-based theoretical model to explain biodiversity in microbial communities, focusing on how antibiotic production and degradation promote coexistence. The core claim is that the producer-sensitive-degrader (PSD) motif, as previously analyzed, is critical for enabling coexistence among microbes. By eliminating redundant (symmetric) topologies, the authors reduce the complexity of possible configurations and aim to identify exact rules that allow for stable coexistence in communities producing and degrading antibiotics.

This approach is significant for the field because it seeks to formalize and generalize the principles underlying microbial diversity, potentially guiding experimental and synthetic community design.

**Strengths**

- The manuscript addresses an important question: what structural features in microbial interaction networks allow for stable coexistence despite antagonistic interactions.
- The use of graph theory to systematically explore the space of possible community interactions could clarify which network motifs are essential for stability.

**Areas for improvement**

- The manuscript lacks a clear and concise statement of the "exact rules" needed for stability. While the PSD motif's importance is discussed, the criteria for stability in more complex networks are not clearly articulated. For example:
  - What fraction of antibiotics in the network must have a PSD motif?
  - Must every configuration be cyclic, and what does “cyclic” mean in this context? Though examples of this cyclicity are presented I think a proper definition is needed, and from it to derive the “magnitude” of cyclicity needed as a rule.
  - How do complexity and symmetry of configurations affect stability?
- The methods and parameter choices are not sufficiently detailed, making it difficult to assess the robustness of the findings. For example:
  - How is stability defined and quantified? For a given topology are eigenvalues searched on multiple parameter sets?
  - What makes one configuration more stable than another? Is it just a matter of having a larger attractor, or still being stable in the face of extensions?
  - What is the range of N and M being studied here? In the text both are not larger than 3 but in figure 2 it seems N can reach to 5. Are there any constraints such has M<=N?
  - What are the values of parameters scanned?
- Line 169- “for any parameter value- all strains have equal abundance at equilibrium” is a very strong statement and I’m not sure I understand its source.
- Table 1’s purpose and message are somewhat unclear. While the manuscript text refers to it as holding a “complete record,” the legend describes it as listing “notable extensions,” which creates some confusion. Additionally, the table only presents stable extensions for strains, and unstable extensions for antibiotics and without clearly stating why. For greater clarity and completeness, I recommend the authors (1) clarify in both the text and the legend what Table 1 is intended to show, (2) provide quantification or at least a summary of the number of possible extension of which how many are stable/unstable, and (3) consider whether these extensions follow distinct rules that could be discussed (4) clearly explain the importance of the bolded resilient to permutation entries. Finally, the information may be more effectively conveyed as a figure or schematic rather than a table, which could help readers grasp the relationships and extensions at a glance.
- Fig. 4: The legend states that “15 simulations [were] carried for each of 900 parameter sets, varying Kp and Kd values,” but each subplot seems to show only a single value. It appears that the different lines reflect initial lattice configurations, with other parameters fixed. Please clarify the meaning of the 900 parameter sets and how Kp/Kd variation is represented.
- Fig. 4: Including a small schematic of the community, as in previous figures, would also aid reader understanding.
- Regarding the extension section: when a new antibiotic is introduced that doesn’t encompass a producer-sensitive the community stays stable. I wonder if this is true also in the more realistic case where the parameters are not equal to all antibiotics, or if there are any “rules” where this is true in this scenario. In other words- how robust is the community to such additions?

Minor comments:

- Fig A.1 legend: “P, S,D,R where the letters stand for Producer, Sensitive, Degrader, and Sensitive respectively”. Change the second Sensitive to resistant.
- Supplementary row 47 fig A.2 – I would state that matrix 2 & 3 are indeed identical even if identities are important.
- Clarify: in the methods line 437 it is said that: “the costs for each antibiotic are the same”, while in the footnote for line 148 it is said that the assumption of identical metabolic costs is made for convenience but is omitted when stable fixed point disappeared when they two antibiotics have different metabolic costs.
- Clarify: In line 146 it is said that one of 430 distinct communities had stable internal fixed point, but it is not clear if these are different run of the N=3, M=2 with different parameters? Multiple topologies?
- Add to the discussion some acknowledgment regarding synergistic interactions between antibiotics that are not as straight forward as the one described in the model (using the binomial formulation)
- Add to the discussion a sentence regarding the costs of the different functions (production>Degrader etc.). In real microbial interactions the costs of production can be much lower, and condition dependent- for instance if the production only occurs when the density of the species is high enough or it is surrounded by other competing species
- Line 93: what is the definition of a “critical interaction graph”?
- Figure 1: I think the example from Kelsic *et al*. there is a bit too symmetrical and another example (such as in figure 2) should be added.
- Figure 1: Panel C is not very informative for the model understanding, but more for the computational reduction. Consider showing it elsewhere.
- Fig. 1 legend: “linked to equations in section “ - no section is detailed.
- The sentence In lines 312-313 should be rephrased. It is clearly not sufficient to have PSD motifs to every antibiotic.
- A better explanation of the meaning of “these PSD motifs must balance each other” (line 313) would be appropriate- if I understand correctly this is what the authors mean by “cyclicity”?

**Conclusion**

The manuscript addresses a timely and important topic using a promising theoretical framework. However, to maximize its impact and clarity, the authors should provide more explicit statements of their main findings, improve methodological transparency, and better connect their theoretical results to biological reality.
